# Social epidemiology of early adolescent problematic screen use in the United States

**DOI:** 10.1038/s41390-022-02176-8

**Published:** 2022-06-29

**Authors:** Jason M. Nagata, Gurbinder Singh, Omar M. Sajjad, Kyle T. Ganson, Alexander Testa, Dylan B. Jackson, Shervin Assari, Stuart B. Murray, Kirsten Bibbins-Domingo, Fiona C. Baker

**Affiliations:** 1 Division of Adolescent and Young Adult Medicine, Department of Pediatrics, University of California, San Francisco, San Francisco, CA USA; 2 Geisel School of Medicine, Dartmouth College, Hanover, NH USA; 3 Factor-Inwentash Faculty of Social Work, University of Toronto, Toronto, ON Canada; 4 Department of Management, Policy and Community Health, University of Texas Health Science Center at Houston, Houston, TX USA; 5 Department of Population, Family, and Reproductive Health, Johns Hopkins Bloomberg School of Public Health, Johns Hopkins University, Baltimore, MD USA; 6 Department of Family Medicine, College of Medicine, Charles R. Drew University of Medicine and Science, Los Angeles, CA USA; 7 Department of Urban Public Health, Charles R. Drew University of Medicine and Science, Los Angeles, CA USA; 8 Marginalization-related Diminished Returns (MDRs) Research Center, Charles R. Drew University of Medicine and Science, Los Angeles, CA USA; 9 Department of Psychiatry and Behavioral Sciences, University of Southern California, Los Angeles, CA USA; 10 Department of Epidemiology and Biostatistics, University of California, San Francisco, San Francisco, CA USA; 11 Center for Health Sciences, SRI International, Menlo Park, CA USA; 12 School of Physiology, University of the Witwatersrand, Johannesburg, South Africa

## Abstract

**Objective:**

To determine sociodemographic correlates of problematic screen use (social media, video games, mobile phones) among a racially/ethnically and socioeconomically diverse population-based sample of 10–14-year-old early adolescents.

**Study design:**

We analyzed cross-sectional data from the Adolescent Brain Cognitive Development Study (Year 2, 2018–2020; *N* = 8753). Multiple linear regression analyses were used to estimate associations between sociodemographic factors (age, sex, race/ethnicity, primary language, household income, parental education) and adolescent-reported problematic video game (Video Game Addiction Questionnaire), social media (Social Media Addiction Questionnaire), and mobile phone use (Mobile Phone Involvement Questionnaire).

**Results:**

Boys reported higher problematic video game use while girls reported higher problematic social media and mobile phone use. Native American, black, and Latinx adolescents reported higher scores across all problematic screen measures compared to non-Latinx white adolescents. Having unmarried/unpartnered parents was associated with higher problematic social media use. Although higher household income was generally protective against problematic video game use, these associations were weaker for black than white adolescents (*p* for interaction <0.05).

**Conclusions:**

Given the sociodemographic differences in problematic screen use, digital literacy education strategies can focus on at-risk populations, encourage targeted counseling by pediatricians, and adapt family media use plans for diverse backgrounds.

**Impact:**

- While sociodemographic differences in screen time are documented, we examined sociodemographic differences in problematic screen use in a large, diverse sample of early adolescents in the US.
- Boys reported higher problematic video game use while girls reported higher problematic social media and mobile phone use.
- Native American, black, and Latinx adolescents reported higher scores across all problematic screen measures compared to non-Latinx white adolescents.
- Although higher household income was generally protective against problematic video game use, these associations were weaker for black than white adolescents.
- Beyond time spent on screens, pediatricians, parents, and educators should be aware of sociodemographic differences in problematic screen use.

## Introduction

Children and adolescents are increasingly interacting with the world through tablets, smartphones, televisions, and gaming consoles;^1^ 95% of US adolescents have access to a smartphone, and 45% report being online “almost constantly.”^2^ Studies have demonstrated associations between more screen time and higher caloric intake, sedentary behavior, depressive and anxiety symptoms, eating disorders, and a poorer quality of life,^3–5^ although relationships may be nuanced. Beyond time spent on screens, it is important to consider the extent of control over usage and interference with other activities, which could reflect problematic use including addiction and impairments in functioning. In some populations, deleterious outcomes may be the result of problematic screen use,^6,7^ which can span various modalities, including video games, social media, and mobile phones.^8^ Problematic video game use is characterized by an inability to regulate control over gaming, resulting in negative consequences in personal, social, occupational, familial, and other relevant areas of functioning.^9^ Problematic social media use is characterized by a perceived need to be online and constantly connected to technology, as typified by colloquialized phrases such as being “alone together.”^10,11^ While problematic mobile phone use shares some similarities with problematic social media use, it includes a broader range of applications that may be used on a mobile phone (e.g., texting, apps, video chat).^12,13^ Regardless of modality, problematic screen use can lead to negative psychological effects and functioning.^9–13^ Recognizing the prevalence of problematic screen use behaviors and sociodemographic elements associated with children’s interaction with video games, social media, and smartphones is crucial in implementing measures to prevent downstream persistent psychological distress, reduction of physical activity, and increased risk of obesity among groups at higher risk.^14^

Prior literature has demonstrated important sociodemographic disparities in general screen time; however, there is a paucity of literature documenting sociodemographic correlates of problematic screen use. Children who belong to minority groups or groups from lower socioeconomic backgrounds report higher levels of screen time than their white peers or peers from higher socioeconomic backgrounds, respectively.^15,16^ Among a sample of US adolescents, black eighth graders reported higher daily use of video games compared to their white counterparts, even after controlling for socioeconomic status.^17^ Social media use tends to be higher in girls than boys.^16^ One Turkish study investigating social media addiction found associations with age and income.^8^ A study of vocational students in Switzerland demonstrated that problematic mobile phone use was more prevalent in children aged 15–16 compared to 19 years or older.^12^ However, data on sociodemographic associations with problematic screen use in the US has been limited, especially among early adolescents who may be at the greatest risk.

With these apparent gaps in the literature, the purpose of our study was to explore problematic screen use behaviors across a population-based and racially/ethnically and economically diverse sample of US children aged 10–14 years old, considering three different domains: video games, social media, and mobile phones.

## Methods

We conducted a secondary cross-sectional analysis of data from the 2-year follow-up of the Adolescent Brain Cognitive Development (ABCD) Study (4.0 release). The ABCD Study is a longitudinal study (baseline 2016–2018) of health and cognitive development in 11,875 children from 21 recruitment sites across the US. The ABCD Study participants, recruitment, protocol, and measures have previously been described in detail.^18^ Participants were 10–14 years old during the 2-year follow-up, which was conducted between 2018 and 2020. After omitting study participants who were not asked problematic screen use questions due to not reporting video game, social media, or mobile phone use (*n* = 3122), 8753 children remained in the analytic sample (Appendix A). Institutional review board (IRB) approval was received from the University of California, San Diego and the respective IRBs of each study site. Written assent was obtained from participants, and written informed consent was obtained from their caregivers.

### Measures and study variables

#### Dependent variables

##### Video Game Addiction Questionnaire (VGAQ)

The six-question VGAQ was used to assess problematic video game use as reported by adolescents. The questions were modeled after the Bergen Facebook Addiction Scale.^19^ The Bergen Facebook Addiction Scale consists of a unidimensional factor structure questionnaire assessing Facebook addiction, but prior authors have extrapolated its application to broader video game and social media addiction among high school and college students.^20,21^ Example questions include “I play video games so much that it has had a bad effect on my schoolwork or job” and “I feel the need to play video games more and more.” Likert-type scale responses ranged from 1 (never) to 6 (very often). Participants who reported any video game use on weekdays or weekends were asked these items.

##### Social Media Addiction Questionnaire (SMAQ)

The six-question SMAQ was used to assess problematic social media use as reported by adolescents. The questions were also modeled after the Bergen Facebook Addiction Scale.^19^ Examples include “I’ve tried to use my social media apps less but I can’t” and “I’ve become stressed or upset if I am not allowed to use my social media apps.” Likert-type scale responses ranged from 1 (never) to 6 (very often). Participants who reported having at least one social media account were asked these items.

##### Mobile Phone Involvement Questionnaire (MPIQ)

The eight-question MPIQ was designed to assess problematic mobile phone use as reported by adolescents.^22^ Examples include “I interrupt whatever else I am doing when I am contacted on my phone” and “I lose track of how much I am using my phone.” Likert-type scale responses ranged from 1 (strongly disagree) to 7 (strongly agree). A prior study has utilized this questionnaire to assess smartphone dependence with respect to digital multitasking during homework among US high school students.^23^ Participants who reported having mobile phones were asked these items.

##### Screen use

Screen use for the following modalities was determined using adolescents’ self-reported hours of use on a typical weekday and weekend: multi-player gaming, single-player gaming, texting, social media, video chatting, browsing the internet, and watching/streaming movies, videos, or TV.^24^ Total typical daily screen use was calculated as the weighted sum ([weekday average × 5] + [weekend average × 2])/7.

#### Independent variables

Parents reported participants’ sex (male or female), country of birth (US or outside US), primary language (English or non-English), and race/ethnicity (non-Latinx/Hispanic white, non-Latinx/Hispanic black, Native American, Latinx/Hispanic, Asian, or Other) at baseline.

In addition, parents reported highest parent education and household income at Year 2. In order to assess highest parent education, the questionnaire asked, “What is the highest grade or level of school you have completed or the highest degree you have received?” and “What is the highest grade or level of school your partner completed or highest degree they received?” Highest parent education was classified as high school or lower versus college or higher. To assess household income, the questionnaire asked, “What is your total combined family income for the past 12 months? This should include income (before taxes and deductions) from all sources, wages, rent from properties, social security, disability and/or veteran’s benefits, unemployment benefits, workman’s compensation, help from relatives (include child payments and alimony), and so on.” Household income was grouped into two categories reflecting the US median household income: less than $75,000 and $75,000 or more.^25^

### Statistical analyses

Data analyses were performed in 2021 using Stata 15.1 (StataCorp). Multiple linear regression analyses were conducted to estimate cross-sectional associations between sociodemographic factors (age, sex, race/ethnicity, primary language of the child, household income, parents’ highest education, parent marital status) and three forms of problematic screen use (video game, social media, mobile phone), adjusting for the site. We expected variation in the association between income and our outcomes by race/ethnicity based on previous literature demonstrating minorities’ diminished returns (MDRs). These were tested by statistical interaction terms of household income multiplied by race/ethnicity (non-Hispanic white = 0). The coefficients for these interactions could be interpreted as differences in the change in the outcome due to a change in household income if the youth was a racial/ethnic minority compared to white (a positive and significant interaction would indicate MDRs). Some adolescents within the sample were twins or siblings. Sensitivity analyses were conducted including only one sibling per family, and findings did not substantially differ; therefore, we present results from the full sample. Propensity weights were applied to match key sociodemographic variables in the ABCD Study to the American Community Survey from the US Census.^26^

## Results

Table 1 describes sociodemographic characteristics of the 8753 participants included. The analytic sample was approximately matched by sex (47.8% female) and racially and ethnically diverse (44.7% racial/ethnic minority). Table 2 shows responses to the problematic screen use questions. All problematic screen use and screen time measures were significantly correlated (Appendix B). Problematic social media and mobile phone use had the strongest correlation (*r* = 0.59, *p* < 0.001).

**Table 1:** Sociodemographic and screen time characteristics of Adolescent Brain Cognitive Development (ABCD) Study participants (*N* = 8753).

	Mean (SD)/%
*Sociodemographic characteristics*	
Age (years)	12.0 (0.7)
Sex (%)	
Female	47.8
Male	52.2
Race/ethnicity (%)	
White	55.3
Latinx/Hispanic	19.4
Black	15.7
Asian	5.1
Native American	3.2
Other	1.3
Primary language of adolescent (%)	
English	88.9
Non-English	11.1
Household income (%)	
Less than $75,000	48.0
$75,000 and greater	52.0
Parents’ highest education (%)	
High school education or less	14.9
College education or more	85.1
Parent marital status (%)	
Married/partnered	70.5
Unmarried/unpartnered	29.5
Screen time	
Total recreational screen time	7.34 (5.81)
Television	1.68 (1.83)
Videos	1.48 (1.93)
Single-player video games	1.07 (1.69)
Multi-player video games	1.20 (1.84)
Texting	0.75 (1.59)
Social media	0.76 (1.68)
Video chat	0.51 (1.37)
Browsing the internet	0.37 (0.80)
Problematic screen use measures	
Video Game Addiction Questionnaire Score^a^	2.09 (1.08)
Social Media Addiction Questionnaire Score^b^	1.85 (0.90)
Mobile Phone Involvement Questionnaire Score^c^	3.10 (1.12)

ABCD propensity weights were applied based on the American Community Survey from the US Census.

*SD* standard deviation.

^a^Asked among a subset who reported video game use (*n* = 7595).

^b^Asked among a subset who reported social media use (*n* = 5652).

^c^Asked among a subset who reported mobile use (*n* = 7361).

**Table 2:** Problematic video game, social media, and mobile phone use in the Adolescent Brain Cognitive Development (ABCD) Study.

Video Game Addiction Questionnaire^a^	Never	Very rarely	Rarely	Sometimes	Often	Very often	
I spend a lot of time thinking about playing video games	27.0%	18.3%	13.3%	22.3%	10.9%	8.2%	
I feel the need to play video games more and more	47.9%	15.5%	15.8%	11.9%	4.8%	4.0%	
I play video games so I can forget about my problems	54.9%	11.6%	9.3%	12.3%	5.9%	6.0%	
I’ve tried to play video games less but I can’t	62.2%	12.5%	9.2%	9.1%	3.6%	3.5%	
I’ve become stressed or upset if I am not allowed to play video games	60.5%	15.8%	8.3%	9.0%	3.2%	3.2%	
I play video games so much that it has had a bad effect on my schoolwork or job	78.5%	10.4%	4.9%	3.9%	1.3%	1.0%	
Social Media Addiction Questionnaire^b^	Never	Very rarely	Rarely	Sometimes	Often	Very often	
I spend a lot of time thinking about social media apps or planning my use of social media apps	35.6%	24.8%	17.1%	16.1%	4.6%	1.8%	
I feel the need to use social media apps more and more	55.3%	17.4%	15.7%	8.2%	2.4%	1.1%	
I use social media apps so I can forget about my problems	61.9%	11.5%	8.3%	11.5%	4.0%	2.9%	
I’ve tried to use my social media apps less but I can’t	62.6%	12.9%	9.1%	9.1%	3.9%	2.5%	
I’ve become stressed or upset if I am not allowed to use my social media apps	68.4%	12.7%	7.2%	7.3%	2.3%	2.2%	
I use social media apps so much that it has had a bad effect on my schoolwork or job	78.8%	9.8%	5.3%	4.2%	1.3%	0.7%	
Mobile Phone Involvement Questionnaire^c^	Strongly disagree	Disagree	Somewhat disagree	Neither agree nor disagree	Somewhat agree	Agree	Strongly agree
I interrupt whatever else I am doing when I am contacted on my phone	17.7%	26.3%	14.3%	11.2%	19.5%	7.8%	3.3%
I often use my phone for no particular reason	13.6%	23.1%	9.3%	10.1%	22.4%	15.8%	5.7%
I feel connected to others when I am using my phone	12.0%	19.1%	9.9%	15.6%	21.8%	16.7%	5.0%
Arguments have arisen with others because of my phone use	32.4%	33.0%	8.0%	9.3%	10.4%	4.9%	2.1%
I lose track of how much I am using my phone.	15.0%	19.6%	9.3%	8.6%	19.8%	17.0%	10.7%
I often think about my phone when I am not using it	28.5%	34.7%	9.8%	7.7%	9.6%	6.1%	3.7%
I have been unable to reduce my phone use	25.3%	36.0%	10.0%	11.6%	8.3%	6.1%	2.7%
The thought of being without my phone makes me feel distressed	44.0%	32.2%	5.9%	6.6%	5.9%	3.2%	2.2%

ABCD propensity weights were applied based on the American Community Survey from the US Census.

^a^Asked among a subset who reported video game use (*n* = 7595).

^b^Asked among a subset who reported social media use (*n* = 5652).

^c^Asked among a subset who reported mobile use (*n* = 7361).

Table 3 shows linear regression analyses examining sociodemographic associations with problematic video game, social media, and mobile phone use. On average, boys reported higher scores for problematic video game use while girls reported higher scores for problematic social media and mobile phone use. Black adolescents reported higher scores for problematic screen use compared to white adolescents across the three modalities. Native American and Latinx/Hispanic adolescents reported higher scores for problematic video game and mobile phone use compared to white adolescents. Asian adolescents reported higher scores for problematic video game use compared to white adolescents. Lower income was associated with higher problematic screen use scores, while lower parent education was associated with higher problematic video game use. Adolescents whose parents were unmarried/unpartnered reported higher problematic mobile phone use scores compared to adolescents whose parents were married/partnered.

**Table 3:** Sociodemographic associations with problematic screen use in the Adolescent Brain Cognitive Development (ABCD) Study.

	Video games^a^		Social media^b^		Mobile phones^c^	
Sociodemographic characteristics	*B* (95% CI)	*p*	*B* (95% CI)	*p*	*B* (95% CI)	*p*
Age	0.02 (–0.02 to 0.05)	0.281	**0.09 (0.04 to 0.13)**	**<0.001**	**0.18 (0.14 to 0.23)**	**<0.001**
Sex						
Female	reference		reference		reference	
Male	**0.74 (0.69 to 0.79)**	**<0.001**	**–0.11 (–0.16 to –0.05)**	**<0.001**	**–0.18 (–0.24 to –0.12)**	**<0.001**
Race/ethnicity						
White	reference		reference		reference	
Latinx/Hispanic	**0.12 (0.04 to 0.20)**	**0.004**	0.06 (–0.04 to 0.15)	0.250	**0.11 (0.004 to 0.21)**	**0.041**
Black	**0.17 (0.10 to 0.25)**	**<0.001**	**0.11 (0.02 to 0.19)**	**0.018**	**0.21 (0.12 to 0.31)**	**<0.001**
Asian	**0.14 (0.04 to 0.25)**	**0.008**	0.02 (–0.12 to 0.15)	0.814	–0.16 (–0.32 to 0.00)	0.050
Native American	**0.25 (0.12 to 0.38)**	**<0.001**	0.14 (–0.06 to 0.33)	0.167	**0.29 (0.11 to 0.47)**	**0.002**
Other	0.14 (–0.12 to 0.40)	0.281	0.04 (–0.40 to 0.49)	0.848	–0.03 (–0.51 to 0.45)	0.902
Primary language of adolescent						
Non-English	reference		reference		reference	
English	0.06 (–0.04 to 0.16)	0.219	0.03 (–0.09 to 0.14)	0.648	–0.11 (–0.23 to 0.02)	0.092
Household income						
$75,000 and greater	reference		reference		reference	
Less than $75,000	**0.17 (0.11 to 0.23)**	**<0.001**	**0.12 (0.05 to 0.20)**	**0.001**	**0.15 (0.07 to 0.23)**	**<0.001**
Parents’ highest education						
College education or more	reference		reference		reference	
High school education or less	**0.10 (0.02 to 0.18)**	**0.014**	0.01 (–0.08 to 0.10)	0.774	0.06 (–0.04 to 0.16)	0.228
Parent marital status						
Married/partnered	reference		reference		reference	
Unmarried/unpartnered	0.03 (–0.03 to 0.09)	0.286	0.07 (–0.00 to 0.14)	0.054	**0.10 (0.02 to 0.18)**	**0.011**

Bold indicates *p* < 0.05. ABCD propensity weights were applied based on the American Community Survey from the US Census.

All models include age, sex, race/ethnicity, primary language, household income, parent education, parent marital status, and site.

^a^Asked among a subset who reported video game use (*n* = 7595).

^b^Asked among a subset who reported social media use (*n* = 5652).

^c^Asked among a subset who reported mobile use (*n* = 7361).

We conducted additional linear regression analyses examining sociodemographic associations with problematic video game use stratified by income given evidence of significant effect modification by income (*p* < 0.05). There was no evidence of significant effect modification by income for problematic social media or mobile phone use (*p* > 0.05). There were some notable differences by race/ethnicity and income level. For black adolescents (compared to white adolescents), those in high-income households reported a *B* = 0.32 (95% CI 0.18–0.45) higher problematic video game use score; however, in low-income households, the difference in problematic video game use score was not significant (*B* = 0.07; 95% CI –0.05 to 0.19).

## Discussion

In this demographically diverse sample of 10–14-year-old early adolescents in the United States, we found multiple noteworthy sociodemographic factors associated with problematic screen use. Boys reported higher problematic video game use scores while girls reported higher problematic social media and mobile phone use scores. Native American, black, and Latinx/a adolescents reported higher problematic video game and mobile phone use measures compared to white adolescents. While higher household income was generally protective against problematic screen use, these associations were weaker for black than white adolescents, which is in line with the expected MDRs theory.^27,28^

Our finding of sex differences in problematic screen use reflects general screen use trends. Boys on average spend more time playing video games than girls, while girls on average spend more time on social media and texting than boys, which was previously shown in the baseline ABCD cohort.^16^ Significant sex differences occurred in all domains of problematic screen use, but the effect was most prominent in problematic video game use. These differences could be informed by children facing increased pressure to conform to culturally sanctified gender roles from an early age.^29^ Furthermore, while boys spend less time on social media than girls,^16^ they may simply foster social connections through different means (i.e., video games instead of social media). Prior studies support this phenomenon such that boys begin to identify themselves as gamers in early adolescence, when gender disparities typically increase.^29,30^ Our findings extend known sex differences in time spent on screens to problematic screen use.

Across the video game, social media, and mobile phone categories, black adolescents reported higher problematic screen use than white adolescents. Native American and Latinx/Hispanic adolescents reported higher problematic video game and mobile phone use compared to white adolescents. Adolescents from lower-income households reported higher problematic screen use. Racial and socioeconomic differences may be explained by residential differences. Black and Latinx/Hispanic parents are more likely to be essential workers and therefore may have less supervision time for their children.^31^ Certain neighborhood environments intrinsically have fewer opportunities for outdoor physical activity in predominantly minority neighborhoods.^32^ Negative perceptions of safety within a neighborhood have been associated with decreased physical activity and increased screen time.^33,34^ Race/ethnicity and socioeconomic status may be a proxy for context and place,^35^ which may narrow options for recreational activities.

Socioeconomic disparities could be partially explained by “escapism,” or the tendency to seek relief and distraction from realities perceived as being unpleasant, especially by entertaining or engaging in a fantasy.^36–38^ For instance, multiple studies demonstrate that video game addiction may permit the user to escape from reality.^36,39^ Prior studies have identified escaping through video games as a preference for virtual over real-life stimuli, and this was associated with higher engagement in video game usage as assessed through an Affect Misattribution Procedure.^39^ While video gaming can be a nonproblematic leisure pursuit or even a passion for many, it becomes problematic when children use video games as a potential coping strategy.^39^ The questions asked in the VGAQ suggest a strong component of problematic coping, potentially exacerbated by psychological escapism. While speculative, this may be associated with the higher problematic video game use among children of parents with lower formal education and lower household income.

Interestingly, income modified race and sex disparities in problematic video game use. In high-income households, there were greater disparities in problematic video game use for black compared to white adolescents, relative to low-income households. This may reflect MDRs, a phenomenon where higher socioeconomic status does not remove disparities between black and white children.^40^ For instance, black adolescents in high-income households experience higher odds of racism than black adolescents in low-income households, compared to white adolescents.^41^ These MDRs then can be measured at the level of function and structure of the brain, and become a vehicle for the trans-generational transition of poverty and low socioeconomic status.^27,28^

Other factors that could explain disparities in problematic screen use are parent–child interactions, where lower socioeconomic status is associated with fewer parent–child interactions, assessed by the frequency of field trips and screen-free conversations.^42^ Parents underestimate adolescents’ social media use.^43^ Heavy parent screen use has been shown to predict child screen use and can lead to distracted parenting, also known as “technoference” or “technology interference.”^44^ Technology interference may be influenced by parental education status, household income, and race/ethnicity.

There are several limitations and strengths of this study worth noting. Directions of causality are unable to be determined due to the cross-sectional nature of the study. Although several potential confounding variables were controlled for, there is a possibility of residual confounders. Measures were also self-reported, which increased subjectivity to reporting and recall bias. Social media and mobile phone behavior may also include a certain degree of overlap.^45^ It is also important to note that the effect sizes of some sociodemographic factors were relatively small. The possibility of selection bias may be exemplified by participants with missing data more likely to be ethnic/racial minorities, born outside of the US, and from lower socioeconomic strata (Appendix A). The strengths of this study are derived from the diverse, large, population-based sample. Furthermore, the novel measures captured contemporary problematic screen use behaviors.

Our findings have significant public health, policy, and clinical implications, particularly to inform the adaptation and implementation of existing video game, social media, and mobile phone guidance for children. This research may further inform targeted screen-related guidance for clinicians, educators, and parents. The American Academy of Pediatrics advocates for a family media use plan, which could be individualized based on some of the disparities in problematic screen use noted in this study. Studies show that parental oversight is critical in adolescence, so educating and informing parents on the warning signs of problematic screen use could be helpful.^46^ Moreover, school and community-level efforts implemented to engage families of color may incorporate tailoring of culturally pertinent messages, mobilizing social networks, and building community coalitions to facilitate culturally competent guidance.^47^ One example would be the decision for communities and schools to provide interested parents with guidance on education and novel research to develop and modify their own family media use plans.

This study represents an advance in our understanding of video game, social media, and mobile phone use among early adolescents, and how problematic patterns of usage are associated with sociodemographic factors. These factors should be incorporated into guidance and policies to individualize efforts on counseling and implementation. Greater knowledge of risk and protective factors for problematic video game, social media, and mobile phone behavior can strengthen our preventive strategies by informing future children-focused or family-based interventions across numerous technological platforms while individualizing the approaches for children in this age range. Comprehension of the social epidemiology of problematic video game, social media, and mobile phone use is crucial, especially given the unprecedented rise of technology usage during the coronavirus disease 2019 pandemic.^48^ Future research may integrate randomized control trials, prospective studies, and individual interviews to discern lifestyle factors that are disproportionately associated with problematic video game, social media, and mobile phone use.

## Supplementary information

Supplementary Information

## Data Availability

Data used in the preparation of this article were obtained from the ABCD Study (https://abcdstudy.org), held in the NIMH Data Archive (NDA). Investigators can apply for data access through the NDA (https://nda.nih.gov/).
