# Correction: Social epidemiology of early adolescent problematic screen use in the United States

**DOI:** 10.1038/s41390-022-02311-5

**Published:** 2022-09-22

**Authors:** Jason M. Nagata, Gurbinder Singh, Omar M. Sajjad, Kyle T. Ganson, Alexander Testa, Dylan B. Jackson, Shervin Assari, Stuart B. Murray, Kirsten Bibbins-Domingo, Fiona C. Baker

**Affiliations:** 1 Division of Adolescent and Young Adult Medicine, Department of Pediatrics, University of California, San Francisco, San Francisco, CA USA; 2 Geisel School of Medicine, Dartmouth College, Hanover, NH USA; 3 Factor-Inwentash Faculty of Social Work, University of Toronto, Toronto, ON Canada; 4 Department of Management, Policy and Community Health, University of Texas Health Science Center at Houston, Houston, TX USA; 5 Department of Population, Family, and Reproductive Health, Johns Hopkins Bloomberg School of Public Health, Johns Hopkins University, Baltimore, MD USA; 6 Department of Family Medicine, College of Medicine, Charles R. Drew University of Medicine and Science, Los Angeles, CA USA; 7 Department of Urban Public Health, Charles R. Drew University of Medicine and Science, Los Angeles, CA USA; 8 Marginalization-related Diminished Returns (MDRs) Research Center, Charles R. Drew University of Medicine and Science, Los Angeles, CA USA; 9 Department of Psychiatry and Behavioral Sciences, University of Southern California, Los Angeles, CA USA; 10 Department of Epidemiology and Biostatistics, University of California, San Francisco, San Francisco, CA USA; 11 Center for Health Sciences, SRI International, Menlo Park, CA USA; 12 School of Physiology, University of the Witwatersrand, Johannesburg, South Africa

Correction to: *Pediatric Research* 10.1038/s41390-022-02176-8, published online 29 June 2022

In the original article, some of the descriptive screen time measures in Table 1 were underestimated and have been corrected. The data of the correlation table in Appendix B have also been updated to reflect these changes. The original article has been corrected.

**Table 1:** Sociodemographic and screen time characteristics of Adolescent Brain Cognitive Development (ABCD) Study participants (*N* = 8753).

	Mean (SD)/%
*Sociodemographic characteristics*	
Age (years)	12.0 (0.7)
Sex (%)	
Female	47.8
Male	52.2
Race/ethnicity (%)	
White	55.3
Latinx/Hispanic	19.4
Black	15.7
Asian	5.1
Native American	3.2
Other	1.3
Primary language of adolescent (%)	
English	88.9
Non-English	11.1
Household income (%)	
Less than $75,000	48.0
$75,000 and greater	52.0
Parents’ highest education (%)	
High school education or less	14.9
College education or more	85.1
Parent marital status (%)	
Married/partnered	70.5
Unmarried/unpartnered	29.5
Screen time	
Total recreational screen time	7.34 (5.81)
Television	1.68 (1.83)
Videos	1.48 (1.93)
Single-player video games	1.07 (1.69)
Multi-player video games	1.20 (1.84)
Texting	0.75 (1.59)
Social media	0.76 (1.68)
Video chat	0.51 (1.37)
Browsing the internet	0.37 (0.80)
Problematic screen use measures	
Video Game Addiction Questionnaire Score^a^	2.09 (1.08)
Social Media Addiction Questionnaire Score^b^	1.85 (0.90)
Mobile Phone Involvement Questionnaire Score^c^	3.10 (1.12)

ABCD propensity weights were applied based on the American Community Survey from the US Census.

*SD* standard deviation.

^a^Asked among a subset who reported video game use (*n* = 7595).

^b^Asked among a subset who reported social media use (*n* = 5652).

^c^Asked among a subset who reported mobile use (*n* = 7361).

**Appendix B:** Correlations among problematic screen use and screen time

		1	2	3	4	5	6	7	8	9	10	11
1	Video Game Addiction Questionnaire Score	–										
2	Social Media Addiction Questionnaire Score	0.44	–									
3	Mobile Phone Involvement Questionnaire Score	0.34	0.59	–								
4	Total recreational screen time	0.31	0.3	0.36	–							
5	Television or videos	0.11	0.13	0.17	0.59	–						
6	Videos	0.25	0.17	0.22	0.66	0.34	–					
7	Single-player video games	0.32	0.13	0.14	0.62	0.34	0.42	–				
8	Multi-player video games	0.38	0.11	0.14	0.62	0.28	0.44	0.52	–			
9	Texting	0.04	0.21	0.25	0.53	0.32	0.29	0.25	0.19	–		
10	Social media	0.03	0.33	0.30	0.54	0.32	0.29	0.23	0.18	0.63	–	
11	Video chat	0.05	0.18	0.20	0.46	0.26	0.25	0.24	0.22	0.49	0.48	–
12	Browsing the internet	0.14	0.16	0.15	0.42	0.27	0.32	0.26	0.22	0.27	0.24	0.20

All *p* < 0.001.

