# Supplementary material for: Social epidemiology of early adolescent problematic screen use in the United States
Source: Pediatr Res. 2022 Jun 29;92(5):1443–9. doi: 10.1038/s41390-022-02176-8 (PMC9243697; doi:10.1038/s41390-022-02176-8)
Supplement: Supplementary file 1 — Supplementary Information [file 41390_2022_2176_MOESM1_ESM.docx]

| Appendix A. Comparison of participants included vs excluded | | | |
| --- | --- | --- | --- |
| **Sociodemographic characteristics** | Included (n=8,753) | Excluded (n= 3,122) | p |
| Sex |  |  | <0.001 |
| Female | 47.8% | 51.7% |  |
| Male | 52.2% | 48.3% |  |
| Race/ethnicity (%) |  |  | <0.001 |
| White | 55.3% | 44.6% |  |
| Latinx / Hispanic | 19.4% | 22.1% |  |
| Black | 15.7% | 21.9% |  |
| Asian | 5.1% | 6.6% |  |
| Native American | 3.2% | 3.0% |  |
| Other | 1.3% | 1.9% |  |
| Primary language (%) |  |  | <0.001 |
| English | 88.9% | 85.3% |  |
| Non-English | 11.1% | 14.7% |  |
| Household income (%) |  |  | 0.009 |
| Less than $75,000 | 48.0% | 42.3% |  |
| $75,000 and greater | 52.0% | 57.7% |  |
| Parents' highest education (%) |  |  | <0.001 |
| High school education or less | 14.9% | 22.7% |  |
| College education or more | 85.1% | 77.3% |  |
| Parent marital status (%) |  |  | 0.003 |
| Married/partnered | 70.5% | 66.1% |  |
| Unmarried/unpartnered | 29.5% | 33.9% |  |
| Participants who did not report video game, social media, or mobile phone use were not asked problematic screen use questions and were excluded. ABCD propensity weights were applied based on the American Community Survey from the US Census. | | | |

| Appendix B. Correlations among problematic screen use and screen time | | | | | | | | | | | | | | |
| --- | --- | --- | --- | --- | --- | --- | --- | --- | --- | --- | --- | --- | --- | --- |
|  |  | 1 | 2 | 3 | 4 | 5 | 6 | 7 | 8 | 9 | 10 | 11 | |  |
| 1 | Video Game Addiction Questionnaire Score | - |  |  |  |  |  |  |  |  |  |  | |  |
| 2 | Social Media Addiction Questionnaire Score | 0.44 | - |  |  |  |  |  |  |  |  |  | |  |
| 3 | Mobile Phone Involvement Questionnaire Score | 0.34 | 0.59 | - |  |  |  |  |  |  |  |  | |  |
| 4 | Total recreational screen time | 0.31 | 0.3 | 0.36 | - |  |  |  |  |  |  |  | |  |
| 5 | Television or videos | 0.11 | 0.13 | 0.17 | 0.59 | - |  |  |  |  |  |  | |  |
| 6 | Videos | 0.25 | 0.17 | 0.22 | 0.66 | 0.34 | - |  |  |  |  |  | |  |
| 7 | Single-player video games | 0.32 | 0.13 | 0.14 | 0.62 | 0.34 | 0.42 | - |  |  |  |  | |  |
| 8 | Multi-player video games | 0.38 | 0.11 | 0.14 | 0.62 | 0.28 | 0.44 | 0.52 | - |  |  |  | |  |
| 9 | Texting | 0.04 | 0.21 | 0.25 | 0.53 | 0.32 | 0.29 | 0.25 | 0.19 | - |  |  | |  |
| 10 | Social media | 0.03 | 0.33 | 0.30 | 0.54 | 0.32 | 0.29 | 0.23 | 0.18 | 0.63 | - |  | |  |
| 11 | Video chat | 0.05 | 0.18 | 0.20 | 0.46 | 0.26 | 0.25 | 0.24 | 0.22 | 0.49 | 0.48 | - | |  |
| 12 | Browsing the internet | 0.14 | 0.16 | 0.15 | 0.42 | 0.27 | 0.32 | 0.26 | 0.22 | 0.27 | 0.24 | 0.20 | |  |
| All p < 0.001 | | | | | | | | | | | | |  | |
